# Characterization of Molecular Heterogeneity Associated With Tumor Microenvironment in Clear Cell Renal Cell Carcinoma to Aid Immunotherapy

**DOI:** 10.3389/fcell.2021.736540

**Published:** 2021-09-23

**Authors:** Weimin Zhong, Yinan Li, Yichu Yuan, Hongbin Zhong, Chaoqun Huang, Jiwei Huang, Yao Lin, Jiyi Huang

**Affiliations:** ^1^The Fifth Hospital of Xiamen, Xiamen, China; ^2^Department of Nephrology, The First Affiliated Hospital of Xiamen University, Xiamen, China; ^3^Department of Clinical Medicine, Fujian Medical University, Fuzhou, China; ^4^Department of Urology, Second Affiliated Hospital, Zhejiang University School of Medicine, Hangzhou, China; ^5^Department of Urology, Renji Hospital, Shanghai Jiao Tong University School of Medicine, Shanghai, China; ^6^Central Laboratory at the Second Affiliated Hospital of Fujian Traditional Chinese Medical University, Collaborative Innovation Center for Rehabilitation Technology, Fujian University of Traditional Chinese Medicine, Fuzhou, China

**Keywords:** clear cell renal cell carcinoma, molecular subtype, tumor microenvironment, chemo drugs, immunotherapy, immune checkpoint blockade, IR score

## Abstract

Clear cell renal cell carcinoma (ccRCC) is the most common type of kidney cancer and has strong immunogenicity. A systematically investigation of the tumor microenvironment (TME) in ccRCC could contribute to help clinicians develop personalized treatment and facilitate clinical decision-making. In this study, we analyzed the immune-related subtype of ccRCC on the basis of immune-related gene expression data in The Cancer Genome Atlas (TCGA, *N* = 512) and E-MTAB-1980 (*N* = 101) dataset, respectively. As a result, two subtypes (C1 and C2) were identified by performing non-negative matrix factorization clustering. Subtype C1 was characterized by increased advance ccRCC cases and immune-related pathways. A higher immune score, stromal score, TMB value, Tumor Immune Dysfunction and Exclusion (TIDE) prediction score, and immune checkpoint genes expression level were also observed in C1. In addition, the C1 subtype might benefit from chemotherapy and immunotherapy. The patients in subtype C2 had more metabolism-related pathways, higher tumor purity, and a better prognosis. Moreover, some small molecular compounds for the treatment of ccRCC were identified between the two subtypes by using the Connectivity Map (CMap) database. Finally, we constructed and validated an immune-related (IR) score to evaluate immune modification individually. A high IR score corresponded to a favorable prognosis compared to a low IR score, while more advanced tumor stage and grade cases were enriched in the low IR score group. The two IR score groups also showed a distinct divergence among immune status, TME, and chemotherapy. The external validation dataset (E-MTAB-1980) and another immunotherapy cohort (IMvigor 210) demonstrated that patients in the high IR score group had a significantly prolonged survival time and clinical benefits compared to the low IR score group. Together, characterization of molecular heterogeneity and IR signature may help develop new insights into the TME of ccRCC and provide new strategies for personalized treatment.

## Introduction

Clear cell renal cell carcinoma (ccRCC) is a typical subtype of renal cell carcinoma (RCC), which is responsible for 70–80% of RCC cases (Martel and Lara, 2003). It was estimated that, in 2019, there would be 73,820 new cases and 14,770 deaths from ccRCC in America, which seriously affects human health (Siegel et al., 2016; Gao et al., 2020). Currently, the most common treatment strategy is surgical intervention, including laparoscopic partial nephrectomy (LPN) and radical nephrectomy (LN) (Porpiglia et al., 2014; Ahmad and Finelli, 2019). However, about 20∼30% of diagnostic RCC cases are metastatic (Gitlitz and Figlin, 2003; Yang et al., 2012). Moreover, despite early-stage RCC patients achieving a 5-year overall survival (OS) rate of 90%, the 5-year OS rate of metastatic or advance-stage RCC patients was less than 10% (Chaffer and Weinberg, 2011; Hirata et al., 2011). Cumulative studies have shown that targeted therapy is an effective method for the treatment of ccRCC, especially for metastatic cRCC patients (Grüllich, 2018). Targeted agents including axitinib (Escudier and Gore, 2011), pazopanib (Sternberg et al., 2010), sorafenib (Gordon, 2008), and sunitinib (Motzer, 2007) have made some progress in metastatic patients. However, the therapeutics were not effective for all patients and some showed drug resistance (Buchler et al., 2015; Baig et al., 2006). Thus, there is an urgent need to identify some molecular subtypes that may have implications on drug selection.

Tumor immunotherapy is a promising treatment method for tumors and has become a research hotspot in tumor therapy, and its efficacy is tightly associated with the TME (Wang et al., 2020). PD-1/PD-L1 is an important immunotherapy target for the treatment of ccRCC (Motzer et al., 2018). One study discovered that RCC patients with positive PD-L1 expression had a higher overall response rate to PD-1/PD-L1 treatment when compared to negative PD-L1 expression (Topalian et al., 2012). Moreover, dis-regulated genes also had an impact on the TME. Studies have shown that high expression level of HMGB1 in ccRCC can induce IL-10 secretion by T regulatory cells and decrease the anti-tumor activity of CD8+T cells (Li et al., 2017). Tim-3 expression in renal cell carcinoma is associated with the invasion of T cells and was considered as a novel therapeutic target for immunotherapy of ccRCC (Cai et al., 2015).

In the present study, we systematically investigated the immune-related (IR) molecular heterogeneity of ccRCC based on IR gene expression. We identified two subtypes with distinct immune microenvironment characteristics and immunotherapy response. These results lay a foundation for the further study of ccRCC immunotherapy and developing a personalized treatment.

## Materials and Methods

### Dataset Collection and Processing

The gene expression data and corresponding clinical information of ccRCC were downloaded from The Cancer Genomics Atlas (TCGA^1^) and ArrayExpress database (E-MTAB-1980^2^), respectively. Among them, the TCGA dataset (training dataset) consisted of 539 ccRCC samples, and the E-MTAB-1980 dataset (external validation dataset) included 101 ccRCC samples (Sato et al., 2013). To obtain a reliable result, we excluded the samples with a survival time less than 30 days, and finally a total of 613 ccRCC (512 samples retrieved from TCGA, 101 samples retrieved from the E-MTAB-1980 dataset) samples were included in the downstream analysis. Moreover, the IR gene symbol names were retrieved from the Immunology Database and Analysis Portal (ImmPort^3^) database (Bhattacharya et al., 2018).

### Molecular Subtypes Identification

The IR molecular subtype was identified by performing non-negative matrix factorization (NMF) clustering analysis in the “NMF” R package^4^ (Gillis, 2020). Principal component analysis (PCA) was performed to identify the robustness and reliability of the molecular subtype. Gene Set Variation Analysis (GSVA) was applied to identify the molecular function of each subtype (Hänzelmann et al., 2013). The reproducibility of the subtypes in the E-MTAB-1980 and TCGA datasets was identified by using the subclass mapping algorithm.

### Exploration of the Relationship Between Subtype and Immune Cell Infiltration

To assess the immune cell infiltration of ccRCC, we firstly retrieved 28 IR cell gene sets from a previously reported article (Charoentong et al., 2016). We then applied the ssGSEA methods to estimate the relative abundance of each immune cell in ccRCC through using the “GSVA” R package (Hänzelmann et al., 2013). Moreover, the TME including immune score, stromal score, and tumor purity of each patient was calculated using the ESTIMATE algorithm^5^. The divergence between immune cell infiltration level, TME, and molecular subtype was evaluated using Wilcoxon rank sum test analysis.

### Evaluating the Benefit of Each Subtype From Immunotherapy and Four Chemo Drugs

The chemotherapeutic response of each ccRCC sample was evaluated through the online database Genomics of Drug Sensitivity in Cancer (GDSC^6^) (Yang et al., 2016). Four common ccRCC treatment chemo drugs including Sorafenib, Sunitinib, Pazopanib, and Axitinib were selected (Liu et al., 2019). The prediction process was completed by using the “pRRophetic” R package and the half-maximal inhibitory concentration (IC50) of each ccRCC patient was calculated by using the ridge regression algorithm (Geeleher et al., 2014). The parameters were set as default. In addition, we also applied the Tumor Immune Dysfunction and Exclusion (TIDE)^7^ algorithm to evaluate the clinical response of ccRCC (Jiang et al., 2018).

### Small Molecular Compounds Prediction for the Treatment of Clear Cell Renal Cell Carcinoma

To predict the small molecule compounds of ccRCC, we first identified the differentially expressed genes (DEGs) between subtypes C1 and C2 using the “limma” R package with | logFC| > 1 and adjusted *P*-value < 0.05 (Ritchie et al., 2015). Then the DEGs were uploaded to the Connective Map (CMap) database, respectively (Lamb et al., 2006). The small molecule compounds were further predicted based on the enrichment value and *P*-value.

### Gene Ontology Terms and Kyoto Encyclopedia of Genes and Genomes Pathway Enrichment Analysis

To investigate the molecular function of DEGs, we performed gene ontology (GO) and kyoto encyclopedia of genes and genomes (KEGG) enrichment analysis to explore the molecular functions and pathways through the “ClusterProfiler” R package. Significant GO terms or pathways were screened with the cutoff adjusted *P*-value < 0.05 (Yu et al., 2012).

### Construction of Immune-Related Score

Firstly, we used univariate Cox regression analysis to select the prognostic DEGs with a *P*-value < 0.05, and a total of 274 prognostic genes were selected for further analysis. Then, we performed PCA analysis to construct an IR relevant score. PC1 and PC2 were selected as the signature scores. The advantage of this method is that the score is concentrated on the set with the largest block of related (or anti-related) genes, while the weight is not combined with the contribution of the genes tracked by other set members (Zhang et al., 2020):

IR = Σ (PC1_i_ + PC2_i_), where i represent the expression of each prognostic signature gene. To validate the prognostic value and clinical benefit, RNAseq datasets of 348 patients with bladder cancer were retrieved from the IMvigor 210 dataset (Mariathasan et al., 2018).

### Statistical Analysis

All the analyses was carried out in the R/3.6.1 environment. The continuous data were evaluated using the Wilcoxon rank sum test. A log-rank test Kaplan–Meier (KM) curve was applied to evaluate the survival divergence of different subtypes. In addition, the Fisher test was employed to calculated the difference for the categorical data. For all tests, a *P*-value < 0.05 was regarded as statistically significant.

## Results

### Identification of Molecular Subtypes of Clear Cell Renal Cell Carcinoma

**FIGURE 1:**
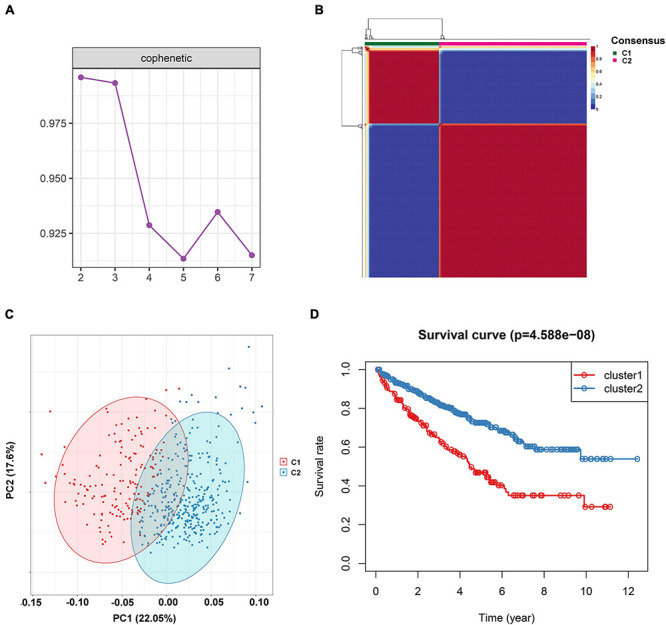
Non-negative matrix factorization clustering analysis to identify potential molecular subtypes of ccRCC based on the immune-related gene expression in TCGA dataset. **(A)** The cophenetic correlation coefficient for the cluster number from 2 to 7. **(B)** Consensus matrix heatmap when *k* = 2. **(C)** Principal component analysis (PCA) for the 512 ccRCC patients, each dot represents a single sample. **(D)** KM survival curve analysis for the overall survival of the two subtypes in ccRCC.

To investigate the IR subtype of ccRCC, we firstly retrieved the reads count data from the TCGA database and transformed it into TPM values. We then used the NMF algorithm to cluster the patients based on the IR gene expression data. To ensure a robust clustering result, we first removed the low expression level genes and subsequently included the selected genes in univariate Cox regression analysis. The genes with a *P*-value less than 0.05 were subjected to NMF clustering analysis in the TCGA dataset. The cophenetic correlation coefficients were applied to determine the optimal *k* value, and *k* = 2 was then selected as the optimal cluster number after a comprehensive consideration (Figure 1A). As shown in Figure 1B, when *k* = 2, we observed that the two subtypes (named C1 and C2) had clear boundaries, suggesting a stable and reliable clustering for the ccRCC sample. PCA was further applied to validate the assignments of the two subtypes. As shown in Figure 1C, the two-dimensional PCA distribution and the two subtypes had similar consistency. The survival curve revealed that C2 had a significantly better survival outcome when compared to C1 in overall survival (*P*-value < 0.001) (Figure 1D). In addition, we also performed NMF clustering analysis in the E-MTAB-1980 dataset, and the clustering result showed two distinct molecular subtypes, which was consistent with the training dataset result (Supplementary Figures 1A–C). The survival difference in the external validation dataset also showed a significant survival difference between the two subtypes (Supplementary Figure 1D) (*P*-value < 0.05). To validate the reproducibility of the molecular subtypes in the TCGA and E-MTAB-1980 datasets, we used the subclass mapping algorithm to compare the expression profiles of the subtypes from the two datasets. As Supplementary Figure 2 shows, we discovered that subtype C1 (*N* = 162) from TCGA was significantly associated with subtype C2 (*N* = 35) from the E-MTAB-1980 dataset, while subtype C2 (*N* = 350) from TCGA was significantly related to subtype C1 (*N* = 66) from the E-MTAB-1980 dataset (Bonferroni-corrected *P*-value = 0.003996).

### Function Enrichment of the Molecular Subtype

To investigate the function of the two subtypes, we performed Gene Set Variation Analysis (GSVA) for the all gene expression datasets based on the subtypes. We then calculated the pathway activity for each sample. We discovered that metabolism-related pathways including fatty acid metabolism, beta alanine metabolism, tryptophan metabolism, and the propanoate metabolism pathway were upregulated in C2, while the p53 signaling pathway, natural killer cell-mediated cytotoxicity, cytokine cytokine receptor interaction, intestinal immune network for IGA production, and primary immunodeficiency pathway were enriched in C1 (Supplementary Figure 3).

### The Relationship Between Molecular Subtype and Tumor Microenvironment

**FIGURE 2:**
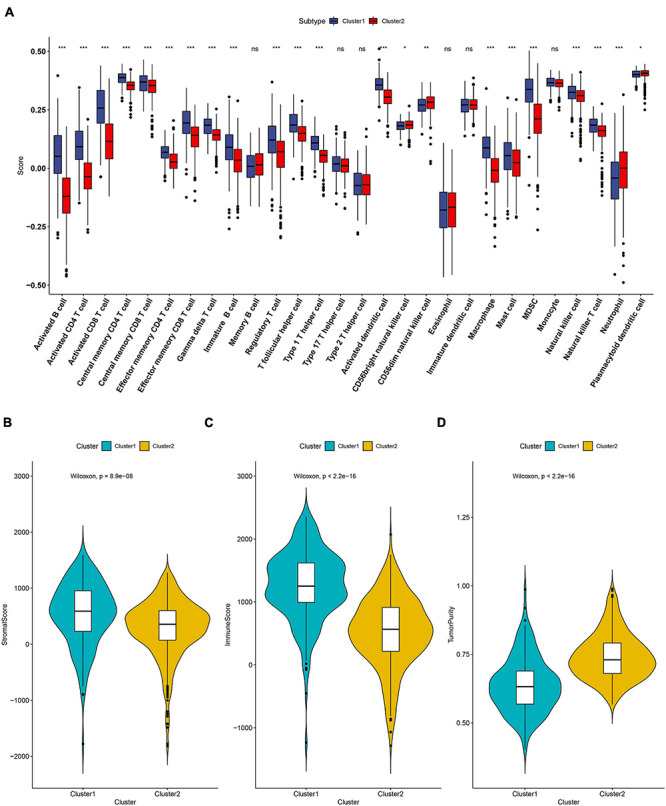
The landscape of immune cell infiltration in the two subtypes. **(A)** Twenty-eight immune cells’ infiltration levels that were calculated by the ssGSEA algorithm in the two subtypes. The comparisons of stromal score **(B)**, immune score **(C)** and tumor purity **(D)** between the two subtypes. ns: not significant, *P < 0.05, **P < 0.01, ***P < 0.001.

The tumor microenvironment (TME) is essential for immune function and has diverse clinical implications for immunotherapy. The divergence of immune cell infiltration and the TME were then explored in the two subtypes. The 28 immune cells’ infiltration level of each ccRCC patient was evaluated by applying the ssGSEA algorithm. As shown in Figure 2A, we observed that the level of activated B cells, CD4 T cells, CD8 T cells, central memory T cells, immature B cells, and T helper cells were significantly high in C1, while CD56dim natural killer cells and neutrophil cells presented at a lower level in C1. Moreover, we also calculated the immune score, stromal score, and tumor purity of each ccRCC sample using the ESTIMATE algorithm. We found that the immune and stromal score were significantly higher in subtype C1 when compared to subtype C2 (Figures 2B,C). However, the tumor purity showed a reverse trend that was lower in C1 (Figure 2D).

### Evaluation of Immunotherapeutic Response in the Clear Cell Renal Cell Carcinoma Subtypes

**FIGURE 3:**
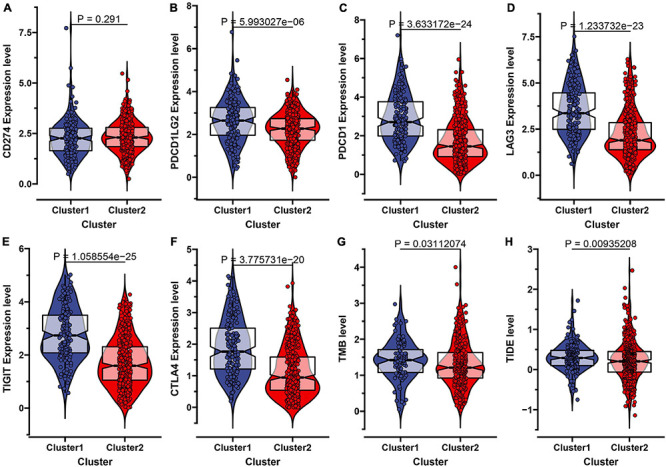
Evaluation of the immunotherapeutic response of the two subtypes in ccRCC. **(A–F)** The expression level of six immune checkpoint inhibitors including CD274 (PD-L1), PDCD1LG2 (PD-L2), PDCD1 (PD-1), LAG3, TIGIT, and CTLA-4 in the two molecular subtypes. **(G)** The comparisons of TMB value between the two subtypes. **(H)** The Tumor Immune Dysfunction and Exclusion (TIDE) prediction score of the two subtypes.

Recently, the immune checkpoint blockade has become a benchmark in the treatment of many tumors. Therefore, we subsequently explored the expression level of some known immune checkpoints including CD274 (PD-L1), PDCD1LG2 (PD-L2), PDCD1 (PD-1), LAG3, TIGIT, and CTLA4. As shown in Figures 3A–F, we found that the PD-L2, PD-1, LAG3, TIGIT, CTLA4 expression levels in subtype C1 were higher than in C2, indicating that patients in subtype C1 with a high expression of immune checkpoint inhibitors were more likely to form an immunosuppressive microenvironment, further leading to tumor immune escape (Dunn et al., 2002). The tumor mutational burden (TMB) has been demonstrated to be an effective predictor of immunotherapy response. In our result, we identified that the TMB level in subtype C2 was significantly lower than C1, which is consistent with a previous report where ccRCC patients’ overall survival corresponded to a low TMB level (Figure 3G; Huang et al., 2021). We also used the TIDE algorithm to estimate immunotherapy response and discovered that the TIDE value in subtype C1 was significantly higher than in C2, indicating that C2 was more likely to respond to immunotherapy (Figure 3H).

### Chemo Drugs for the Treatment of Different Subtypes

**FIGURE 4:**
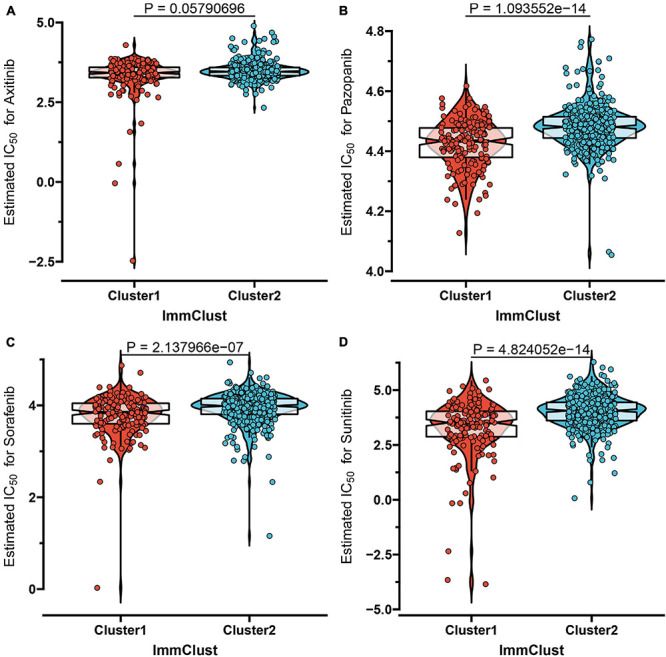
The IC_50_ values of four chemo drugs in the two molecular subtypes of ccRCC including Axitinib **(A)**, Pazopanib **(B)**, Sorafenib **(C)**, and Sunitinib **(D)**.

Despite the fact that the curative effect of chemotherapy is limited in ccRCC, especially in advanced ccRCC, and considering that chemotherapy is the conventional therapy method for ccRCC, we thus attempted to evaluate the response of the two subtypes to four chemo drugs (Axitinib, Pazopanib, Sorafenib, Sunitinib). Therefore, we applied the ridge regression algorithm to train the GDSC cell line dataset, and obtained satisfactory prediction accuracy through 10-fold cross-validation. The IC50 value was estimated for each ccRCC sample according to the predictive model. As shown in Figures 4A–D, apart from Axitinib (*P*-value = 0.058), most of the chemo drugs including Pazopanib (*P*-value = 1.09e-14), Sorafenib (*P*-value = 2.14e-07), and Sunitinib (*P*-value = 4.82e-14) showed a lower IC_50_ value in subtype C1, which indicated that patients in C1 were more likely to respond well to chemo drugs.

### Immunotherapy Response Prediction and Small Molecular Compounds Identification Between the Two Subtypes

**FIGURE 5:**
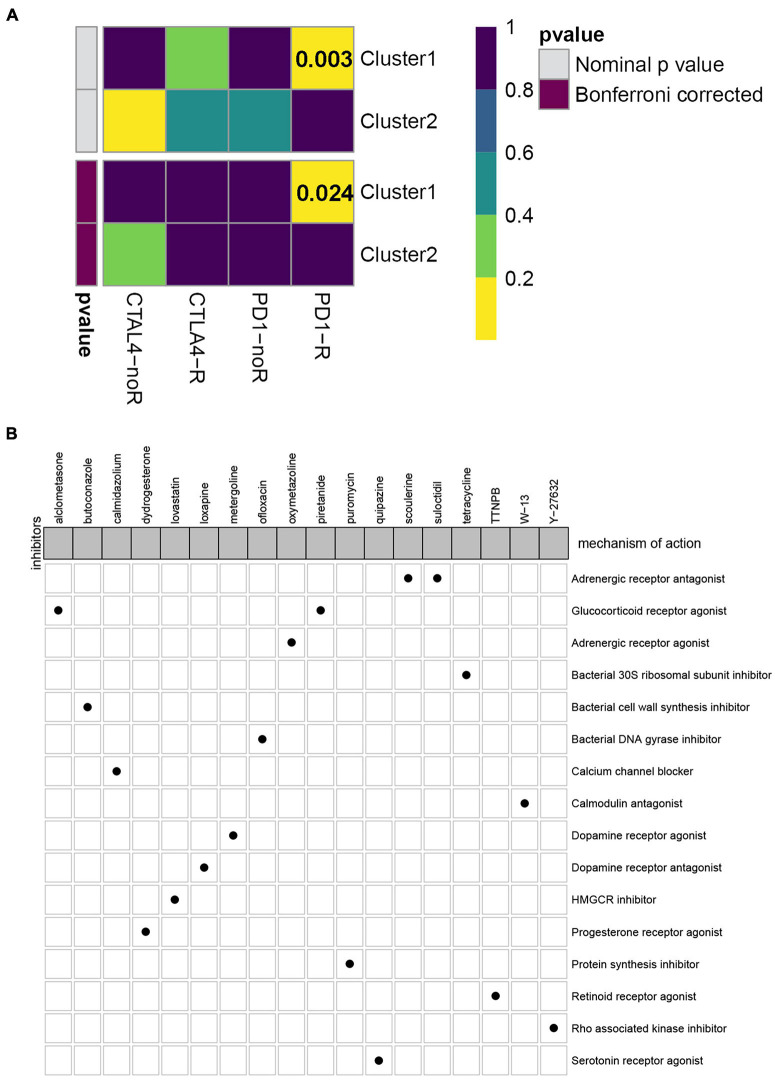
Evaluation of potential compounds and immunotherapy response to the two subtypes. **(A)** Submap analysis demonstrated that cluster 1 had more sensitivity to the programmed cell death protein 1 inhibitor (Bonferroni-corrected, *P* = 0.024). **(B)** CMap database analysis identified candidate drugs targeting the two molecular subtypes based on the DEGs.

Currently, immune checkpoint inhibitors are regarded as the routine drugs for the treatment of advanced ccRCC cases (Parikh and Bajwa, 2020). Here, we investigated the likelihood of response to immunotherapy by using the TIDE algorithm, and it demonstrated that patients in subtype C2 (108/350 = 0.309) were more likely to respond to immunotherapy than those in C1 (25/162 = 0.154) (Fisher exact test *P*-value = 0.0002). In addition to the TIDE prediction, we also compared the expression level of the two subtypes and identified another published dataset that included 47 patients with melanoma that responded to immune checkpoint inhibitors therapy by performing submap mapping analysis (Roh et al., 2017). Considering the worse prognosis of C1, we were delighted to discover that patients in subtype C1 were more sensitive to anti-PD-L1 therapy (Figure 5A). To identify the small molecular compounds of the ccRCC subtypes, we first performed differentially expressed analysis between C1 and C2. A total of 349 DEGs were identified, including 99 upregulated genes and 250 downregulated genes (Supplementary Table 1). These genes were then uploaded to the CMap database and the compounds with an absolute enrichment value more than 0.5 were retained. As a result, CMap mode-of-action (MoA) analysis of the 19 compounds indicated 16 mechanisms of action shared by the above drugs. As shown in Figure 5B, we can observed that scoulerine and suloctidil shared the MoA of adrenergic receptor antagonist, and alclometasone and piretanide shared the MoA of alucocorticoid receptor agonist. These results might provide potential therapeutic targets for the subtypes.

### Generation of Immune-Related Gene Signatures and Functional Annotation

**FIGURE 6:**
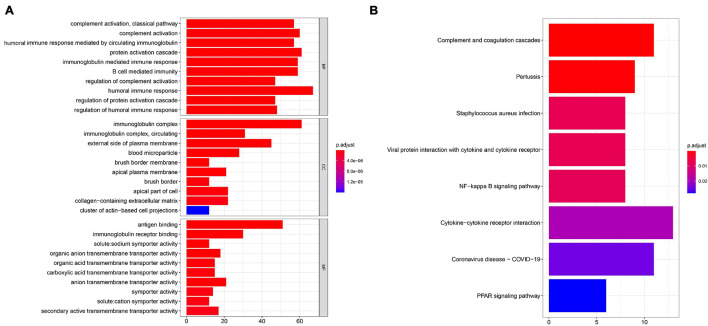
GO **(A)** and KEGG **(B)** enrichment analysis was performed to identify potential function and pathway of DEGs.

**FIGURE 7:**
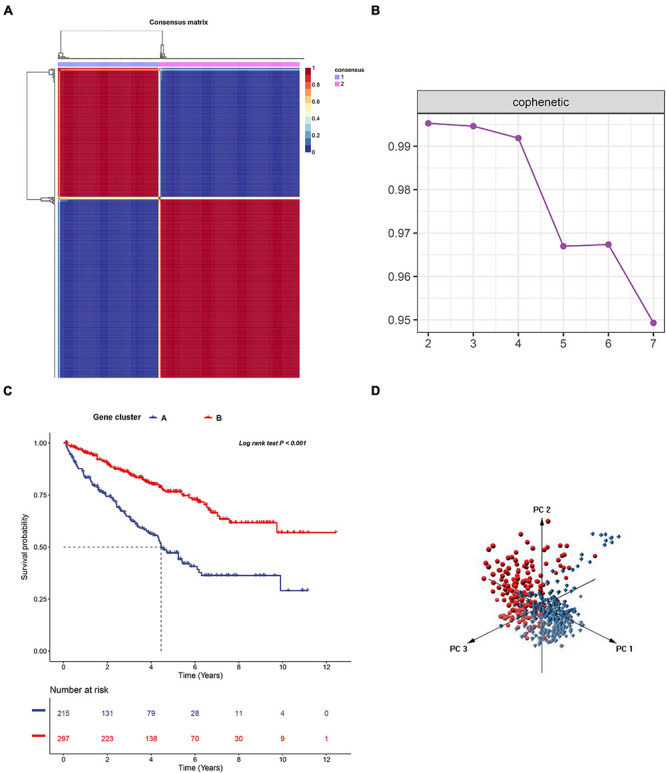
The non-negative matrix factorization (NMF) clustering analysis to identify the genomic subtype of ccRCC based on the 274 DEGs. **(A)** Consensus matrix heatmap when *k* = 2. **(B)** The cophenetic correlation coefficient for the cluster number from 2 to 7. **(C)** KM survival curve analysis for the overall survival of the two subtypes in ccRCC. **(D)** Principal component analysis (PCA) for the 512 ccRCC patients, each dot represents a single sample.

**FIGURE 8:**
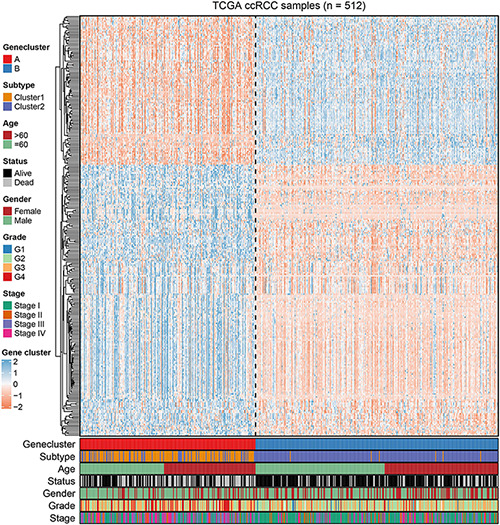
The NMF clustering of DEGs in TCGA cohorts to categorize patients into two genomic subtypes (A and B). The gene clusters, immune-related subtype, tumor stage, age, survival status, gender, grade, and age were termed as patient annotations.

**FIGURE 9:**
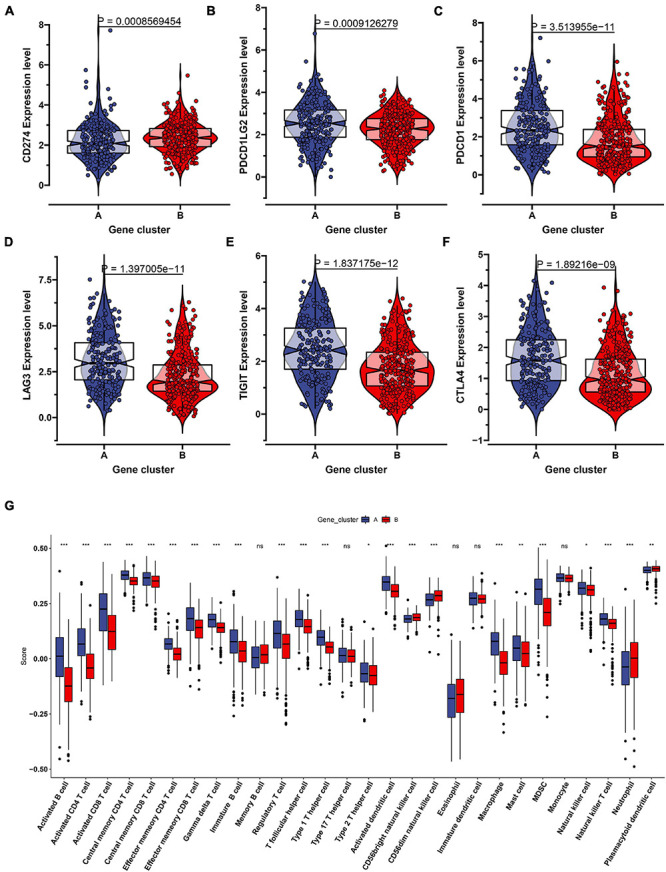
The evaluation of the immune checkpoint and immune cell infiltration in the two genomic clusters. The expression level of six immune checkpoint including CD274 **(A)**, PDCD1LG2 **(B)**, PDCD1 **(C)**, LAG3 **(D)**, TIGIT **(E)**, CTLA4 **(F)** were showed in the two genomic clusters. **(G)** Twenty eight immune cells infiltration level were compared in the two genomic clusters. ns: not significant, *P < 0.05, **P < 0.01, ***P < 0.001.

To further uncover the function role of each molecular subtype, we firstly performed univariate Cox regression analysis on the 349 DEGs, and a total of 274 prognostic DEGs were identified. We then using the “clusterProfiler” R package to conduct GO and KEGG enrichment analysis on the 274 DEGs. As shown in Figure 6A, we discovered that the genes were mainly enriched in complement activation, B cell-mediated immunity, and humoral immune response in the biological processes (Supplementary Table 2). Moreover, the complement and coagulation cascades, NF-kappa B signaling pathway, and cytokine-cytokine receptor interaction were observed in the KEGG pathway enrichment result (Figure 6B and Supplementary Table 3). Interestingly, these GO terms and pathway results were similar to the GSEA results. We further conducted NMF clustering analysis based on the 274 DEGs to categorize patients into different immune genomic subtypes. Interestingly, two immune genomic subtypes (named gene clusters A and B) were identified (Figures 7A,B). The KM curve analysis result suggested that patients in gene cluster B had a better prognosis than A (*P*-value < 0.001) (Figure 7C). In addition, the PCA analysis could completely distinguish ccRCC samples between A and B (Figure 7D). Interestingly, we observed that most of the cases from subtype C2 were in gene cluster A (291/350 = 83.14%), while 96.30% (156/162) of cases from subtype C1 were classified into gene cluster B (Supplementary Figure 4). These results indicated that these DEGs were immune phenotype-related genes and might explain why gene cluster A was relevant to worse survival outcome. We further assessed the relationship between the gene clusters and clinical trait. As shown in Figure 8, we observed that most low-grade cases were enriched in gene cluster B, while advanced stage and grade cases were mainly focused on gene cluster A. Surprisingly, the patients in gene cluster B had large consistency with subtype C1. We also explored the difference of immune checkpoint expression between gene clusters A and B. As expected, except PD-L1 (Figure 9A), we found all immune checkpoints including PD-L2, PD-1, LAG3, TIGIT, and CTLA4 had a lower expression level in gene cluster B (Figures 9B–F). However, the infiltration level of activated B cells, activated CD4 T cells, activated CD8 T cells, central memory T cells, immature B cells, and T helper cells were higher in gene cluster A (Figure 9G).

### Immune Score Construction

**FIGURE 10:**
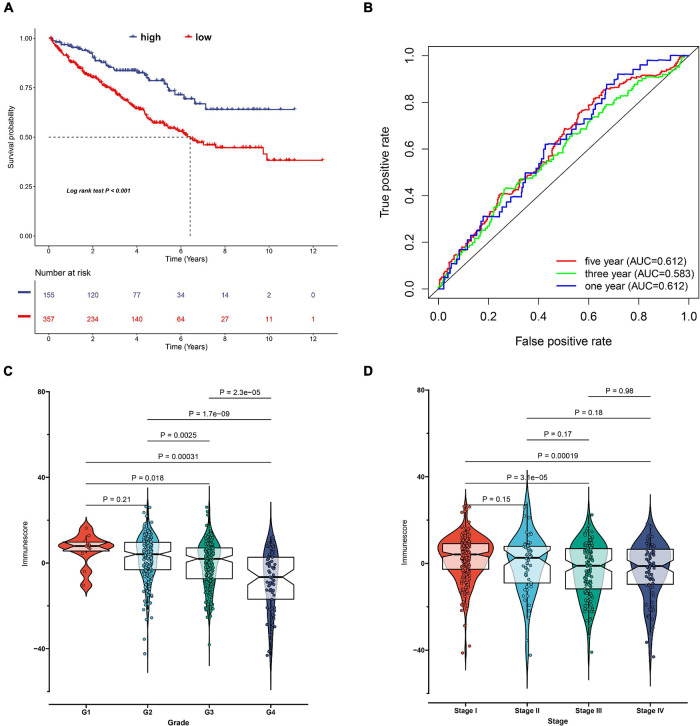
Construction of immune-related (IR) score via principal component analysis. **(A)** Kaplan–Meier curves for patients with high and low IR score subgroups in TCGA cohort. **(B)** The receiver operator curve analysis for the IR score. The relationship between IR score and tumor grade **(C)** and stage **(D)**.

**FIGURE 11:**
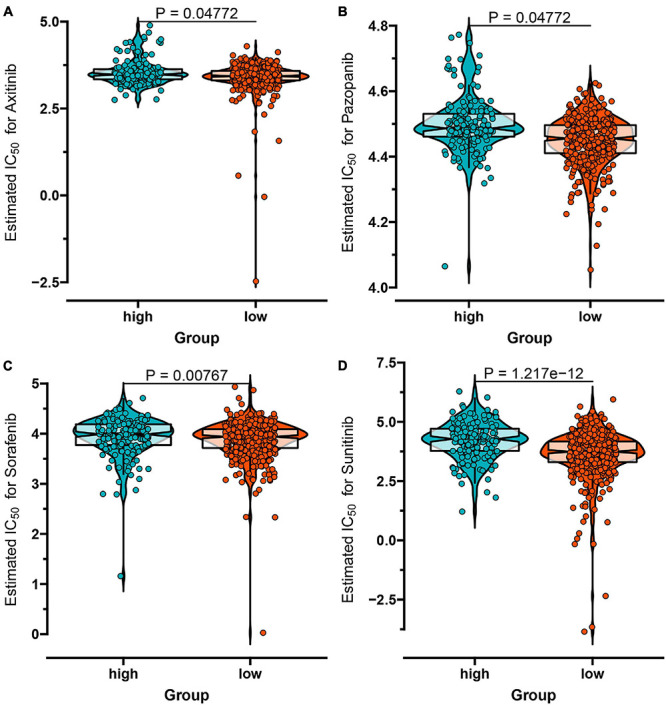
The IC_50_ values of four chemo drugs in the IR score group of ccRCC including Axitinib **(A)**, Pazopanib **(B)**, Sorafenib **(C)**, and Sunitinib **(D)**.

Considering the individual heterogeneity and complexity of IR subtypes, we developed a scoring system to quantify the immune pattern of individual patients with ccRCC based on 274 DEGs, named as the IR score. The KM curve analysis revealed that those with a high IR score had a better survival outcome (Figure 10A). To determine whether IR score can serve as a prognostic predictor, we performed ROC analysis and found that the IR score had the potential capability to ascertain the prognosis of ccRCC (Figure 10B). We further investigated the relationship between IR and clinical trait (grade and stage), and demonstrated that IRS was negatively correlated with grade and stage (Figures 10C,D). We also evaluated whether IR can serve as an independent prognostic factor via Cox regression analysis. As shown in Supplementary Figure 5A, we found that IR was a prognostic factor among the clinical factors in the univariate Cox regression analysis (*P*-value < 0.001), while there was no significant divergence in the multivariate Cox regression analysis (Supplementary Figure 5B). We further evaluated the immune cell infiltration and immune microenvironment, and observed that a high infiltration level of activated B cells, CD4 T cells, CD8 T cells, central memory T cells, immature B cells, and T helper cells was enriched in the low score group, while the level of CD56dim natural killer cells and neutrophils was higher in the high score group (Supplementary Figure 6). In addition, the immune and stromal scores were higher in subtype C1 when compared to subtype C2, whereas the level of tumor purity was low in the IR score group (Supplementary Figures 7A–C). Considering that the curative effect of chemotherapy is limited in ccRCC, we subsequently examined the sensitivity of the four chemo drugs to the IR score group. As shown in Figure 11, we found that the patients in the low IR score group showed more sensitivity to chemo drugs, indicating that the low IR score group may benefit more from chemo drugs. Finally, we identified several small molecular compounds between the low and high IR score groups. Among these compounds, ciprofibrate and clofibrate shared the MoA of PPAR receptor agonist, tiabendazole shared the MoA of angiogenesis inhibitor, and digitoxigenin shared the MoA of ATPase inhibitor (Supplementary Figure 8).

### Validation of the Immune-Related Score and the Role in Predicting Immunotherapeutic Benefits

**FIGURE 12:**
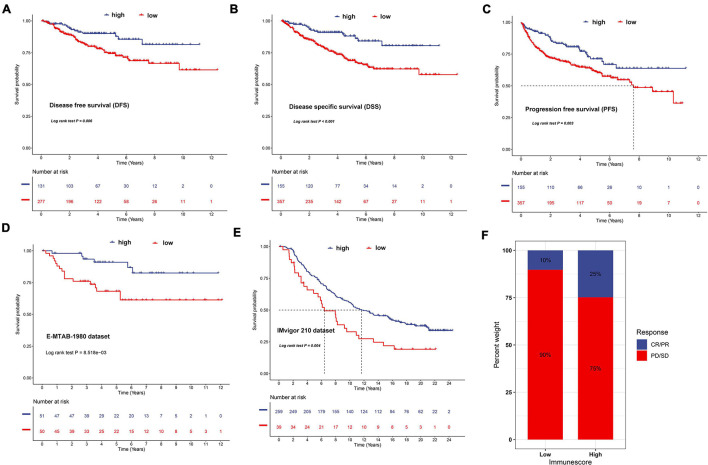
Validation of the prognostic value of IR in disease-free survival **(A)**, disease-special survival **(B)**, and progression-free survival **(C)** of ccRCC, E-MTAB-1980 dataset **(D)**, and IMvigor210 cohort **(E)**, respectively. **(F)** The proportion of patients with response to PD-L1 blockade immunotherapy in low or high IR score groups. Responder/non-responder: 10%/90% in the low IR score groups and 25%/75% in the high IR score groups.

To validate the prognostic value of the IR score, we retrieved the disease-free survival (DFS), disease-specific survival (DSS), and progression-free survival (PFS) datasets of ccRCC. As shown in Figures 12A–C, we discovered that a significant survival difference between the high IR score and low IR score groups existed in these datasets. We also validated it in the external validation dataset, and found that patients in the high IR score group had a better survival outcome (Figure 12D). The immunotherapy represented by PD-L1 and PD-1 blockades has achieved unprecedented success in cancer treatment. We then explored whether the IR score could predict patient response to PD-L1 and CTLA-4 blockade therapy based on IMvigor210. As expected, we were delighted to see that patients with a high IR score showed significant clinical benefits and prolonged survival (Figure 12E). The immune response and positive therapeutic benefits of patients in the distinct IR score group to immune checkpoint blockade treatment was further validated (Figure 12F).

## Discussion

Clear cell renal cell carcinoma is a heterogeneous disease that is associated with a poor prognosis in the advanced stage. How to prolong patient survival time and reduce death in advanced ccRCC remains a difficult clinical problem (Gao et al., 2020). Moreover, ccRCC has strong immunogenicity. ccRCC is characterized by a substantial number of inflammatory cells including NK cells, macrophages, T cells, and NK cells. Immune suppression is usually generated in the TME in advanced ccRCC through multiple mechanisms to avoid immune attack. For example, mechanisms weaken the presentation of effective antigens, reduce the impact of T cells, and promote immune tolerance, which are vital to tumor occurrence and development (Noessner et al., 2012). Thus, the TME plays a crucial role in the development of ccRCC. In recent years, immunotherapy has emerged as a new pillar for the treatment of tumors (Rini et al., 2019). The immune checkpoint blockade has attracted a lot of attention and has achieved remarkable results in immunotherapy, such as the use of PD-1/PD-L1 inhibitors and CTLA-4 inhibitors (Chae et al., 2018). However, the curative effects for some patients are still not satisfactory (Gulati and Vaishampayan, 2020). Several studies have demonstrated that individualized variation may contribute to the phenomenon (Takagi et al., 2020). Thus, exploring and understanding the subtypes of ccRCC will contribute to a better understanding of the heterogeneity of this disease (Jiang et al., 2020).

The ImmPort database collected a large number of known IR genes, which facilitated a comprehensive analysis of the role of IR genes in ccRCC (Bhattacharya et al., 2018). Wan et al. (2019) constructed a risk model to predict the prognosis of ccRCC based on seven IR genes including LAU, ISG15, IRF9, ARG2, RNASE2, SEMA3G, and UCN. Liao et al. (2021) used four prognostic IR genes (CRABP2, LTB4R, PTGER1, and TEK) to establish a gene signature to predict the prognosis of ccRCC. Zou and Hu (2020) developed a 14 IR gene signature to predict clinical outcome of kidney renal clear cell carcinoma. However, these studies mainly focused on the prognostic gene value and did not consider the heterogeneity and complexity of ccRCC. Thus, making a comprehensive analysis of IR subtypes will not only provide new insights into the molecular mechanism of ccRCC but also help us to better develop a personalized treatment in ccRCC. In the present study, we identified two IR molecular subtypes of ccRCC according to the gene expression profile. We found that patients in subtype C2 had a better survival outcome than those in C1. We then compared several known immune checkpoint’s (PD-L1, PD-L2, PD-1, LAG3, TIGIT, IDO1, CTLA-4) expression levels between the two subtypes. We found that most of the immune checkpoint inhibitors had a higher expression level in C1 when compared to C2. The previously studies reported that patients with a high expression level of immune checkpoint genes were more likely to form an immunosuppressive microenvironment and promote tumor immune escape (Dunn et al., 2002). These results suggest that patients in C1 may benefit from immune checkpoint inhibitor therapies. The TME (immune score, stromal score, and immune purity) and immune cell infiltration have been reported to be tightly associated with the prognosis and immunotherapy of cancers (Luo et al., 2020). Thus, we also investigated the relationship between subtype and TME and immune cell infiltration. Interestingly, we found that patients in subtype C1 tended to have a higher expression level of CD8^+^ and CD4^+^ T cell infiltration than those in C2. This indicates that the anti-tumor effect of high T cell infiltration is offset by the strong immunosuppressive pathway activated by over-expressed immune checkpoint proteins (Matsushita et al., 2016; Hua et al., 2020). However, further research is needed to demonstrate the potential molecular interactions between the molecular subtype and the immune cells of tumor immune status in ccRCC. In addition, we also discovered that the immune score and stromal score were higher in C1 when compared to C2. Previous studies have reported that high immune score and stromal score corresponded to poor survival (Luo et al., 2020). These findings were consistent with our results. Similarly, TMB value also showed a similar conclusion with the previous studies that presented a high level in C1, but a lower level in the C2 subtype (Huang et al., 2021).

Cumulative studies have demonstrated that chemo drugs including axitinib, pazopanib, sorafenib, and sunitinib have achieved some progress in advanced ccRCC cases (Koudijs et al., 2018). Here, we investigated the curative effect of four common chemo drugs for the subtypes C1 and C2. Interestingly, most of the IC_50_ values of chemo drugs in the C1 subtype were lower than subtype C2, suggesting that patients in subtype C1 were more sensitive to chemo drugs and may benefit from these drugs. In addition, we also identified 18 small molecular compounds and 16 mechanisms of action for the treatment of the molecular subtypes. These drugs included adrenergic receptor antagonist (scoulerine and suloctidil), glucocorticoid receptor agonist (alclometasone and piretanide), adrenergic receptor agonist (oxymetazoline), protein synthesis inhibitor (puromycin), and HMGCR inhibitor (lovastatin). We also discovered other candidate drugs which might pave the way for implementation of subtype treatments for ccRCC patients.

To further reveal the molecular function and pathway that are involved in the subtypes, we conducted GSVA enrichment analysis for the two subtypes. We found that C2 corresponded to metabolism-related pathway, while C1 was associated with p53 signaling and cytokine-cytokine receptor interaction pathways. Studies have revealed that cytokine-cytokine receptor interaction is an important IR pathway that regulates the interaction between cytokines in tumors and is involved in the development and occurrence of tumors (Lee and Rhee, 2017). Considering that subtype C1 has a poor prognosis and more advanced ccRCC patients, it is reasonable that tumor-related pathways were enriched in subtype C1. In addition, we also performed limma analysis and obtained 274 DEGs between C1 and C2, and pathway enrichment results showed that DEGs mainly focused on the complement and coagulation cascades, NF-kappa B signaling pathway, and cytokine-cytokine receptor interaction, which is similar with our previously result, indicating that these DEGs were regarded as immune phenotype-related gene signatures. Similar to the IR gene clustering results, we also identified two genomic clusters based on these DEGs. The two genomic clusters were significantly related to distinct survival outcomes and TME landscapes. Due to the poor prognosis of ccRCC, more potential and valuable biomarkers are urgently needed. Zhang et al. (2021) developed an IR lncRNA-based model (AC012236.1, AC078778.1, AC078950.1, AC087318.1, and AC092535.4) for survival prediction in ccRCC. Liao et al. (2021) developed and validated the prognostic value of IR genes (CRABP2, LTB4R, PTGER1, and TEK) in ccRCC. Xiang et al. (2021) developed a four IR lncRNA signature for the prognosis of ccRCC through WGCNA analysis and Cox regression analysis. However, these risk models may be unstable and easily influenced by the gene expression level, especially considering the strong immunogenicity and heterozygosity of ccRCC. Thus, to guide therapeutic strategies for individual patients more precisely, we further constructed a IR score to quantify the IR patterns of individual tumors. The IR scores were closely associated with clinical information and can also serve as a prognostic biomarker for ccRCC survival. In addition, the prognostic value and therapeutic benefits of IR score were further validated in an external validation dataset and the IMvigor 210 cohort (Mariathasan et al., 2018).

Although we identified two molecular subtypes and constructed an IR score based on expression profile data of ccRCC, several limitations should be noted. Firstly, the samples with clinical information in the external validation dataset were relatively few and need to be expanded. Besides, due to the absence of an appropriate ICI-based ccRCC dataset, we used the different immunotherapy dataset across different malignancies (urothelial cancer) to validate the effects of IR score. Finally, all the discoveries were based on the dataset analysis and *in vitro* and *in vivo* experiments need to be performed to further verify these results.

In conclusion, we comprehensively investigated IR subtypes among 613 ccRCC samples based on the IR gene expression data, and systematically linked these subtypes with TME characteristics. We also evaluated the difference of chemo drug sensitivity and immunotherapy response between the subtypes. Finally, we developed an IR score to quantify the immune pattern of individual patients with ccRCC. These results may contribute to promoting our understanding of the characteristics of TME infiltration and provide new strategies for personalized treatment.

## Data Availability Statement

The original contributions presented in the study are included in the article/Supplementary Material, further inquiries can be directed to the corresponding author/s.

## Author Contributions

JwH, YaL, and JyH designed the study. CH, YiL, and YY collected the clinical information and gene expression data. WZ and YY analyzed the data. WZ, YiL, and HZ wrote and revised the manuscript. All the authors contributed to the article and approved the submitted version.

## Conflict of Interest

The authors declare that the research was conducted in the absence of any commercial or financial relationships that could be construed as a potential conflict of interest.

## Publisher’s Note

All claims expressed in this article are solely those of the authors and do not necessarily represent those of their affiliated organizations, or those of the publisher, the editors and the reviewers. Any product that may be evaluated in this article, or claim that may be made by its manufacturer, is not guaranteed or endorsed by the publisher.

## Footnotes

1
https://portal.gdc.cancer.gov/

2
https://www.ebi.ac.uk/arrayexpress/

3
https://www.immport.org/

4
https://cran.r-project.org/web/packages/NMF/index.html

5
http://r-forge.r-project.org

6
https://www.cancerrxgene.org/

7
http://tide.dfci.harvard.edu/login/

## Supplementary Material

Supplementary Figure 1 The non-negative matrix factorization (NMF) clustering analysis to identify potential molecular subtypes of ccRCC based on the immune-related gene expression in the E-MTAB-1980 dataset. **(A)** The cophenetic correlation coefficient for the cluster number from 2 to 7. **(B)** Consensus matrix heatmap when *k* = 2. **(C)** Principal component analysis (PCA) for the 101 ccRCC patients, each dot represents a single sample. **(D)** KM survival curve analysis for the two subtypes.

Supplementary Figure 2 Subclass mapping analysis revealed that subtype cluster 1 from TCGA was significantly associated with subtype cluster 2 from the E-MTAB-1980 dataset, while subtype cluster 2 from TCGA was significantly related to subtype cluster 1 from the E-MTAB-1980 dataset (Bonferroni-corrected *P*-value = 0.003996).

Supplementary Figure 3 Gene Set Variant Analysis (GSVA) for the two subtypes on the basis of the 512 samples.

Supplementary Figure 4 Sankey plot showing the changes of immune-related molecular subtypes, gene cluster, IR score, and patient survival status.

Supplementary Figure 5 Identification of the independence of the IR score and clinical information through univariate Cox regression analysis **(A)** and multivariate Cox regression analysis **(B)**.

Supplementary Figure 6 The landscape of immune cells in the two IR score groups.

Supplementary Figure 7 The comparisons of the stromal score **(A)**, immune score **(B)**, and tumor purity **(C)** in the IR score groups.

Supplementary Figure 8 Identification of small molecular compounds by using the CMap drug database between the high IR score and low IR score groups.
