# Supplementary figures and images for: Characterization of Molecular Heterogeneity Associated With Tumor Microenvironment in Clear Cell Renal Cell Carcinoma to Aid Immunotherapy
Source: Front Cell Dev Biol. 2021 Sep 23;9:736540. doi: 10.3389/fcell.2021.736540 (PMC8495029; doi:10.3389/fcell.2021.736540)

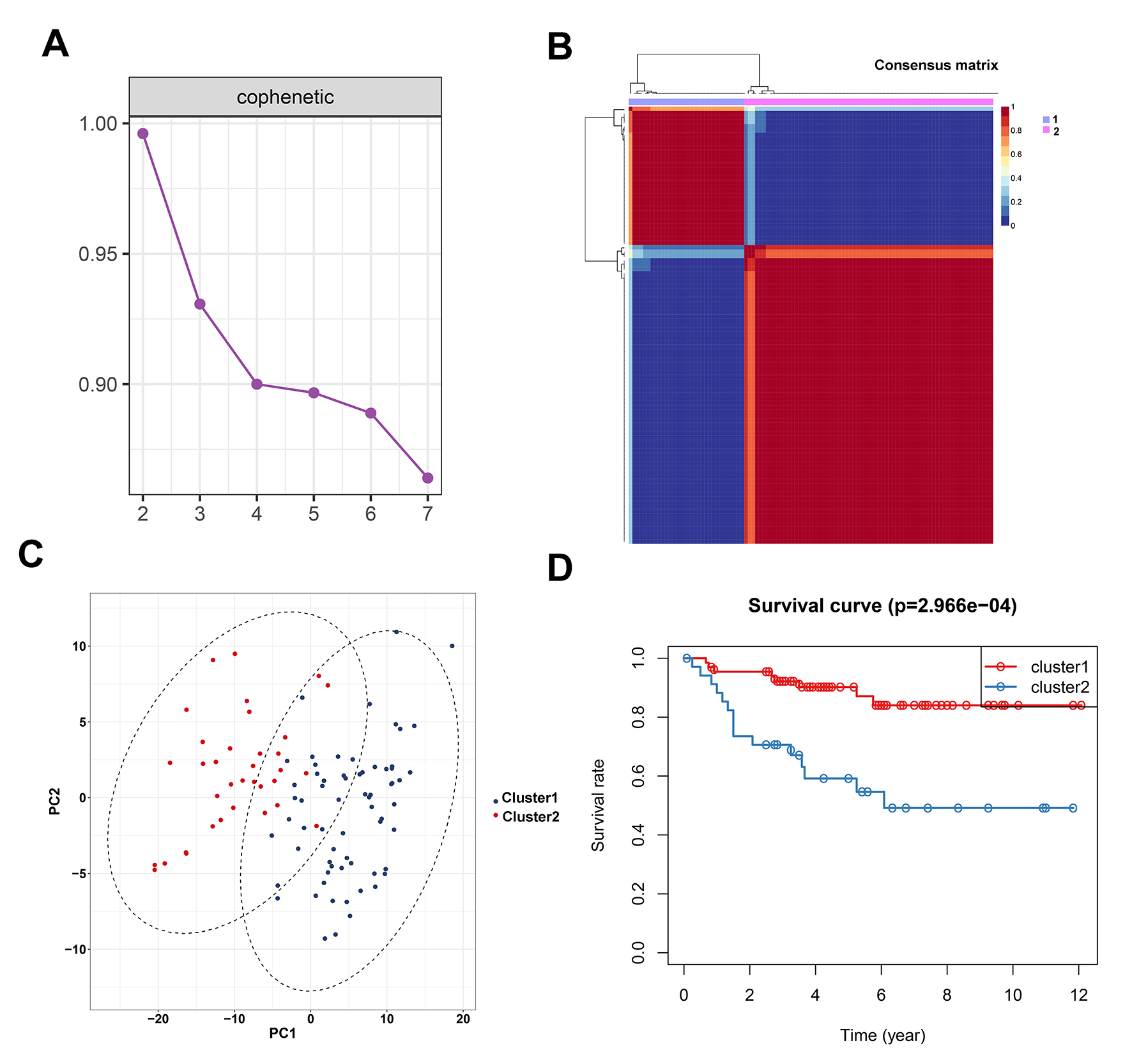

Supplement: Supplementary Figure 1 — The non-negative matrix factorization (NMF) clustering analysis to identify potential molecular subtypes of ccRCC based on the immune-related gene expression in the E-MTAB-1980 dataset. (A) The cophenetic correlation coefficient for the cluster number from 2 to 7. (B) Consensus matrix heatmap when k = 2. (C) Principal component analysis (PCA) for the 101 ccRCC patients, each dot represents a single sample. (D) KM survival curve analysis for the two subtypes. [file Image_1.JPEG]

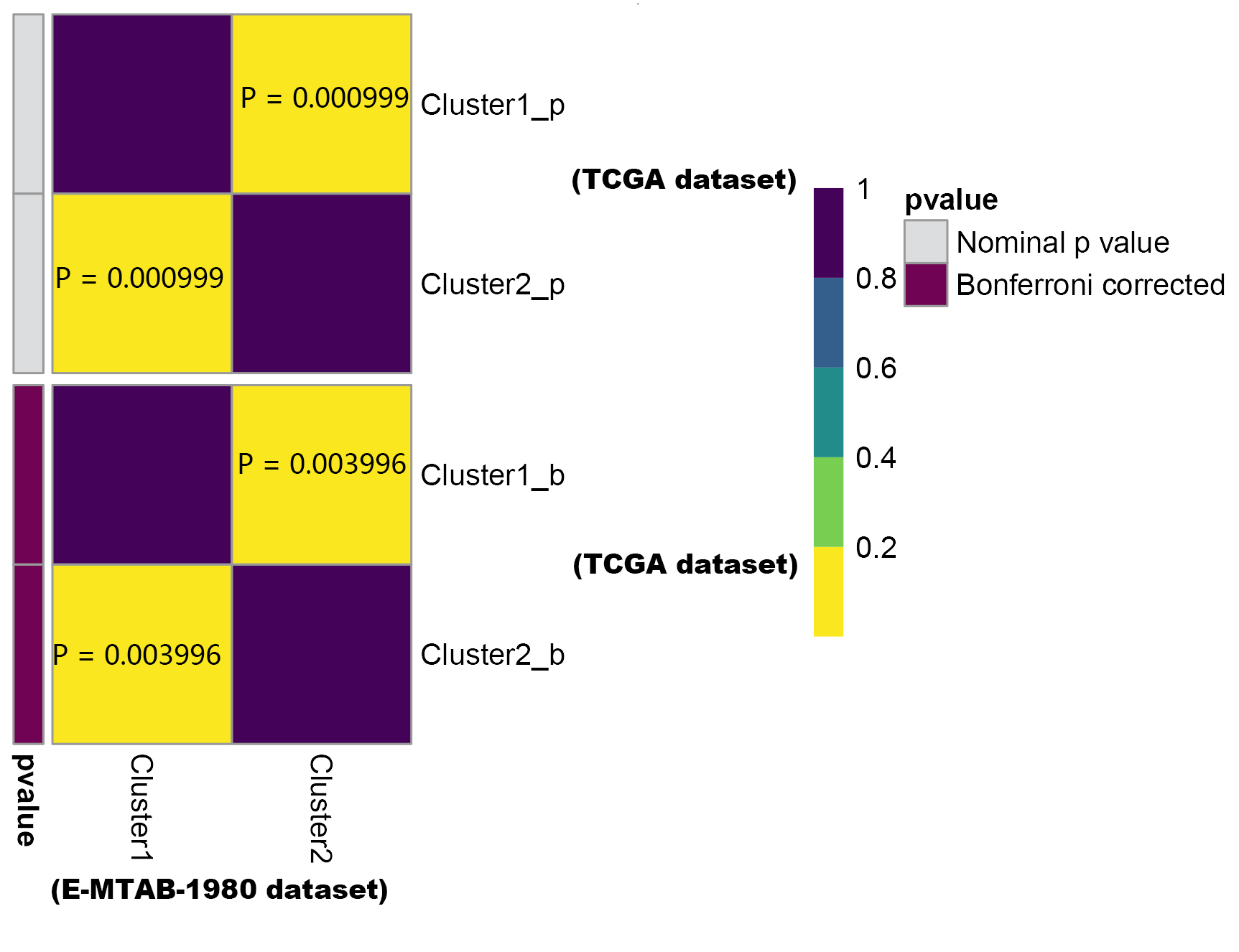

Supplement: Supplementary Figure 2 — Subclass mapping analysis revealed that subtype cluster 1 from TCGA was significantly associated with subtype cluster 2 from the E-MTAB-1980 dataset, while subtype cluster 2 from TCGA was significantly related to subtype cluster 1 from the E-MTAB-1980 dataset (Bonferroni-corrected P-value = 0.003996). [file Image_2.JPEG]

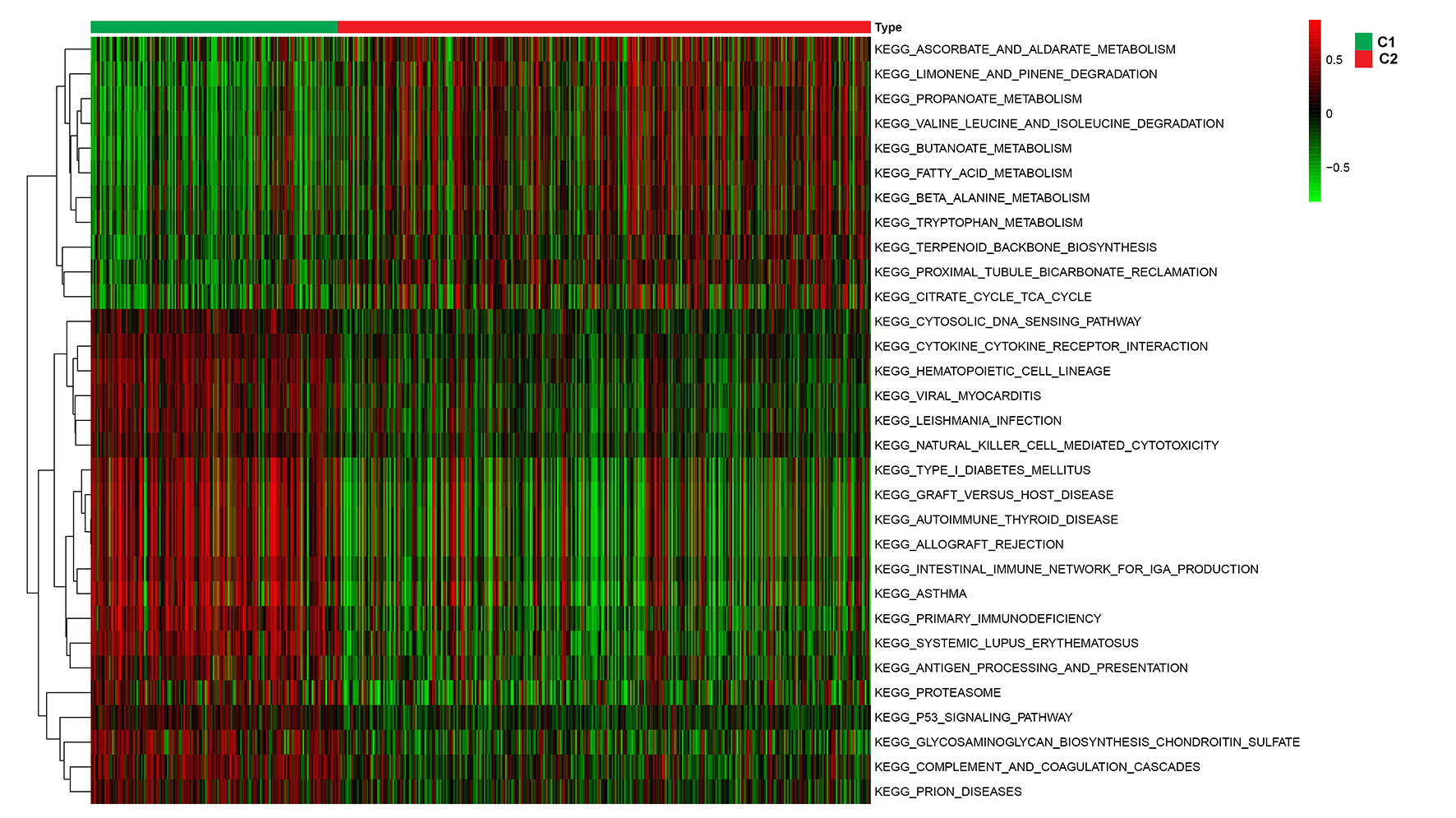

Supplement: Supplementary Figure 3 — Gene Set Variant Analysis (GSVA) for the two subtypes on the basis of the 512 samples. [file Image_3.JPEG]

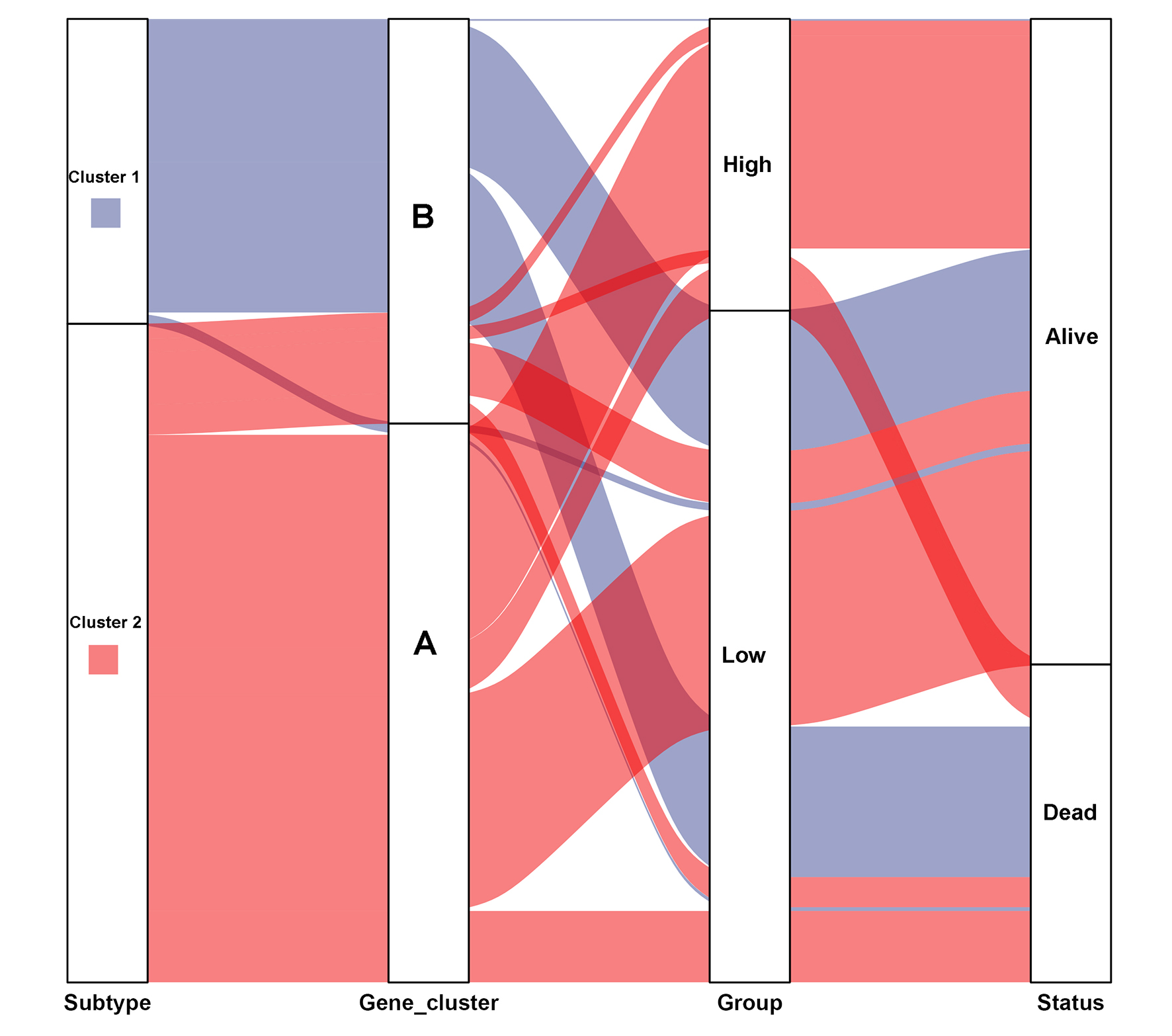

Supplement: Supplementary Figure 4 — Sankey plot showing the changes of immune-related molecular subtypes, gene cluster, IR score, and patient survival status. [file Image_4.JPEG]

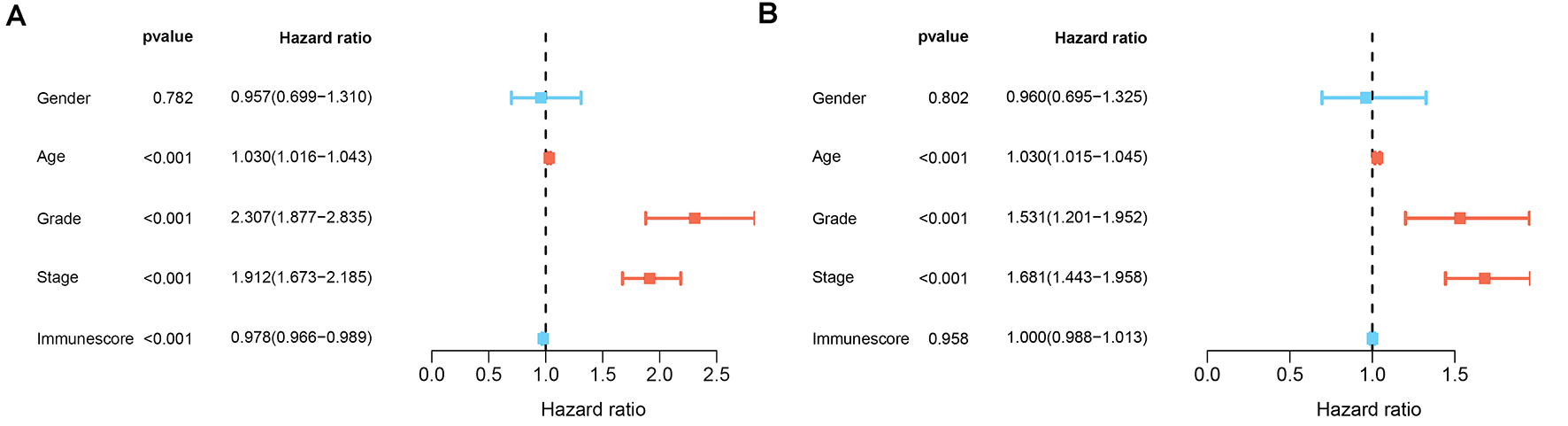

Supplement: Supplementary Figure 5 — Identification of the independence of the IR score and clinical information through univariate Cox regression analysis (A) and multivariate Cox regression analysis (B). [file Image_5.JPEG]

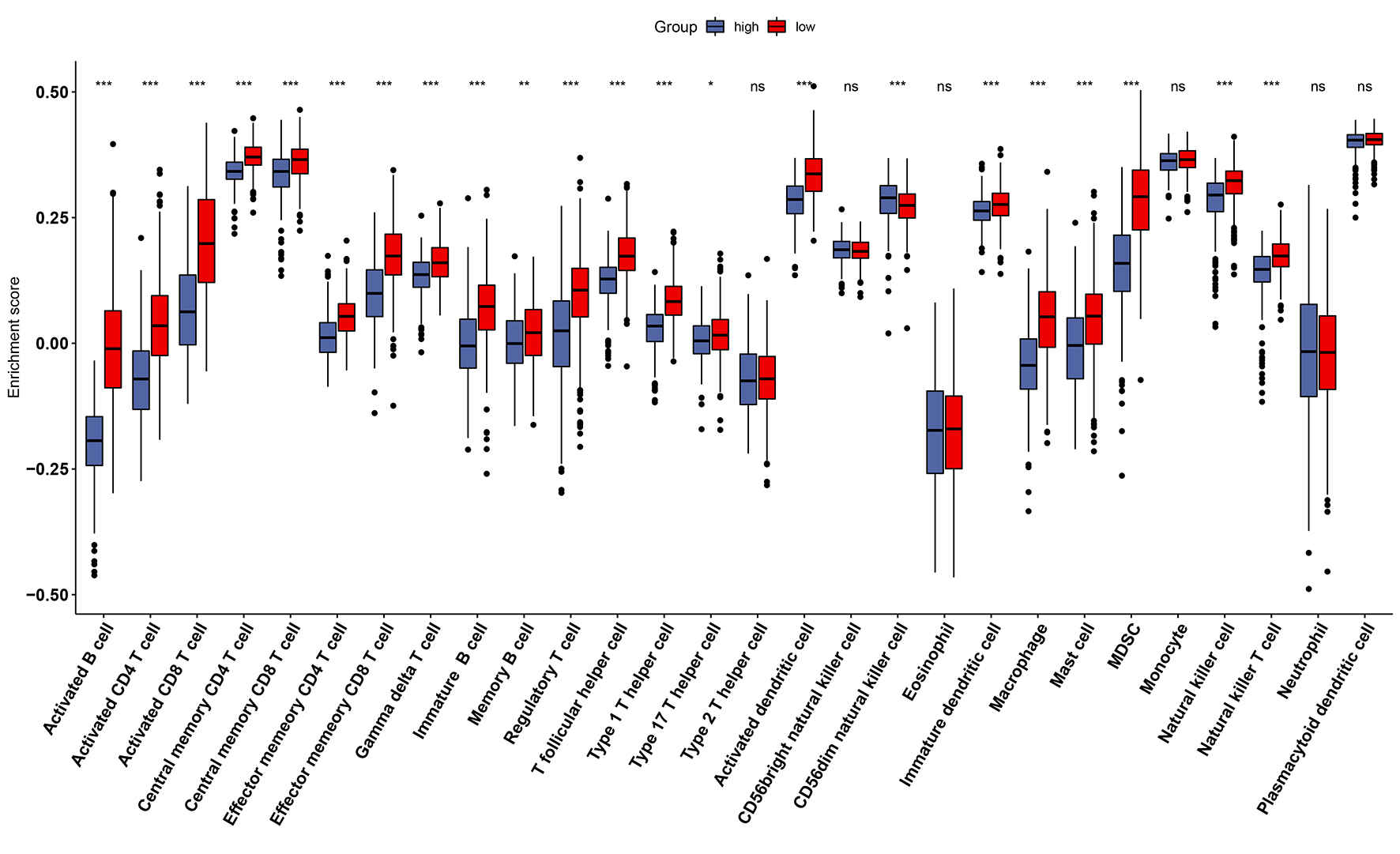

Supplement: Supplementary Figure 6 — The landscape of immune cells in the two IR score groups. [file Image_6.JPEG]

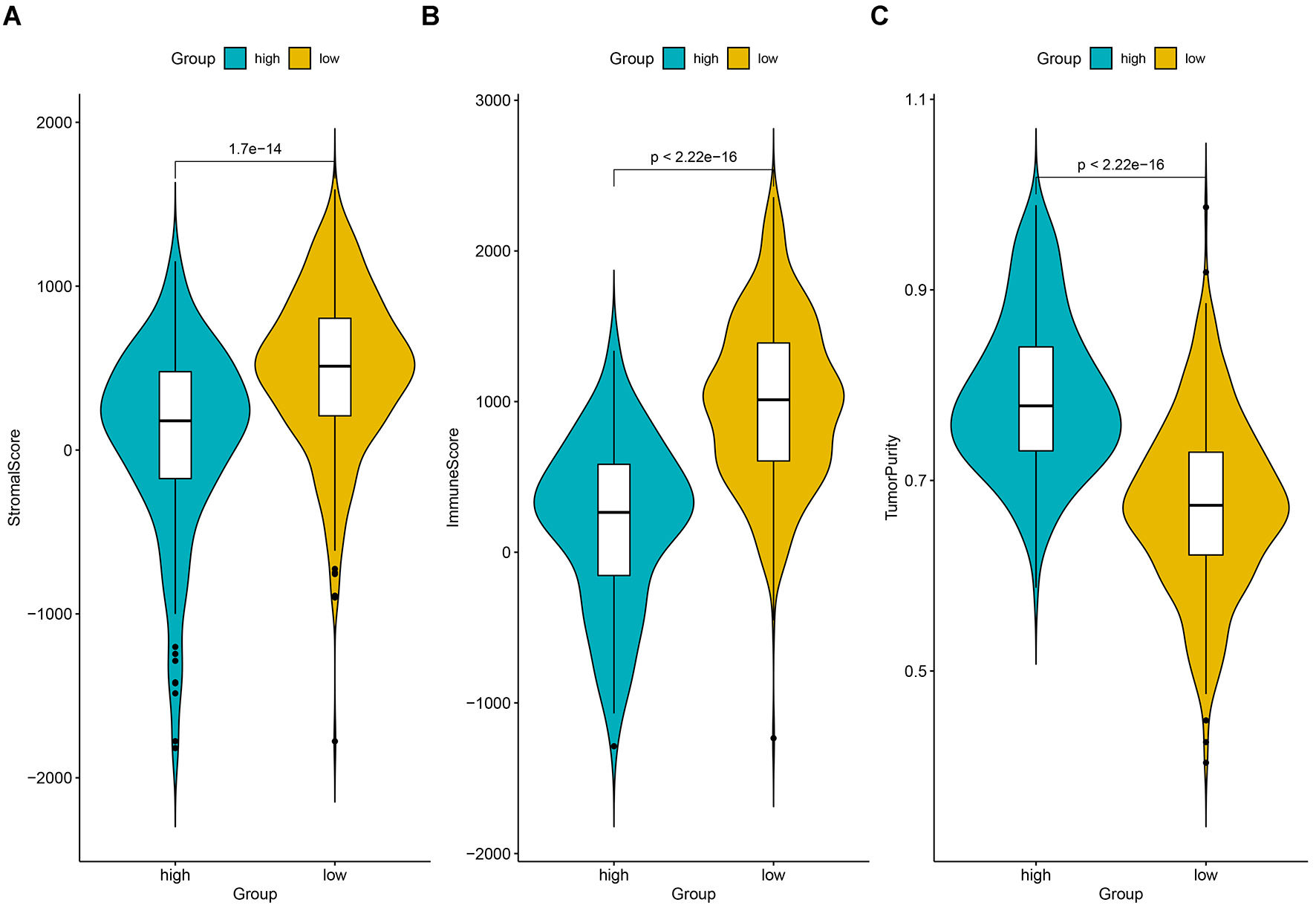

Supplement: Supplementary Figure 7 — The comparisons of the stromal score (A), immune score (B), and tumor purity (C) in the IR score groups. [file Image_7.JPEG]

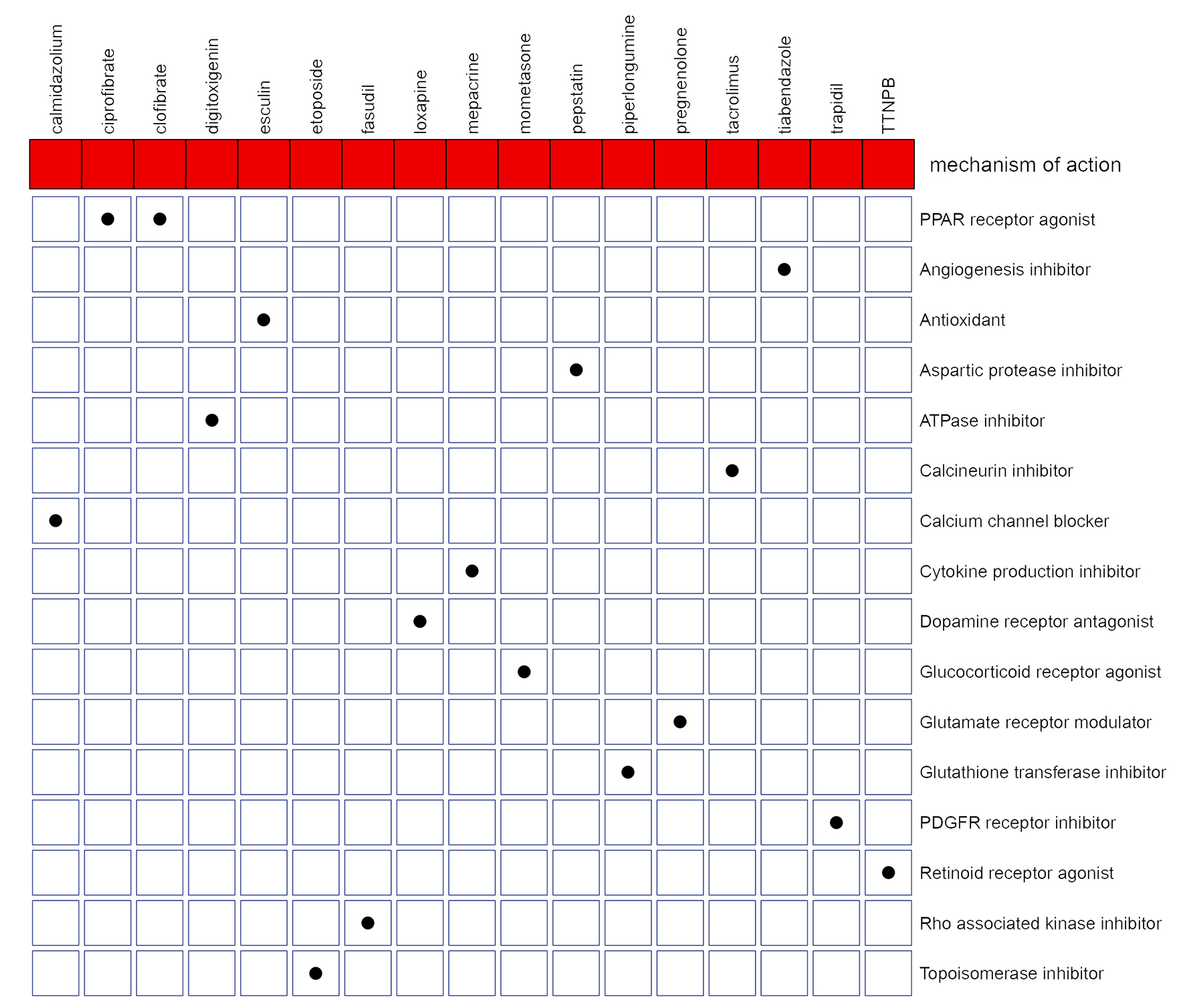

Supplement: Supplementary Figure 8 — Identification of small molecular compounds by using the CMap drug database between the high IR score and low IR score groups. [file Image_8.JPEG]
